# Application of Artificial Intelligence in analysing meteorological data for health research: A rapid review

**DOI:** 10.1007/s11869-026-02085-3

**Published:** 2026-09-05

**Authors:** Lysanne Veerle Michels, Lucy Smith, Suzan Ghannam, Charles Gadd, Hajira Dambha-Miller

**Affiliations:** 1 Primary Care Research Centre, University of Southampton, Southampton, SO16 5ST United Kingdom; 2 Nuffield Department for Women’s & Reproductive Health, University of Oxford, Oxford, United Kingdom

**Keywords:** Artificial intelligence, Predictive modelling, Climate change, Weather, Public health

## Abstract

**Introduction:**

Artificial intelligence (AI) has rapidly advanced as a key analytical tool for processing complex datasets across disciplines, including environmental and health research. Meteorological data is increasingly used to understand and mitigate health risks, often linked to climate change. This rapid review aims to synthesise studies involving AI for meteorological data applications in health research to better understand its use.

**Methods:**

PubMed, Web of Science, and Scopus were systematically searched from 2020 to 2025 following a standardised framework in line with PRISMA-RR guidelines. Eligible studies included any empirical design involving human-related health research where AI techniques were applied to meteorological data. Two reviewers independently screened studies, extracted data, and synthesised findings narratively.

**Results:**

Twelve studies met eligibility criteria. Despite heterogeneity in study design and sample size, most examined the impact of extreme pollution levels and temperature variations on the prevalence and severity of respiratory, bacterial, and other diseases. AI methods primarily included Random Forest models, as well as time-series and clustering analyses. Model performance was commonly evaluated using sensitivity; however, methodological justification was often insufficiently recorded.

**Conclusion:**

Overall, findings suggest that incorporating meteorological variables enhances the prediction of health outcomes, although detailed population characteristics were frequently underreported. This restricts the generalisability and applicability of AI-driven health models incorporating meteorological data, with stronger methodological rigour and clearer reporting standards needed to support reliable future development.

## Background

Meteorological variables such as ambient temperature, humidity, precipitation, atmospheric pressure, and air quality are well established as key determinants of morbidity and mortality and as drivers of healthcare demand worldwide. Air pollution alone contributes to an estimated 4.2 million premature deaths annually, particularly in middle- to low-income countries (World Health Organisation 2024), while mortality risk increases by 10–25% for each degree Celsius rise above regional temperature thresholds (Gasparrini et al. 2015; Mora et al. 2017). A 167% rise in heat-related deaths among adults aged ≥65 years has been observed compared with the 1990 s, exceeding demographic expectations and signalling rapidly worsening exposure and vulnerability associated with climate change (Romanello et al. 2024). Across Europe, an estimated 368,183 heat-related deaths occurred between 2010 and 2022, of which 89% were among people aged ≥65 years (Wu et al. 2025). UK-focused analyses similarly confirm significant heat-related excess mortality, and project further increases as warming continues (Dahil et al. 2025; Dickinson et al. 2025). Children are also at higher risk of weather-induced conditions due to immature thermoregulation (Schapiro et al. 2024), while individuals with mental health conditions or long-term illness are particularly vulnerable (Government UK 2025). In addition to heat, cold spells and flooding disproportionately affect these populations, contributing to cardiovascular stress, respiratory illness, displacement, and disruption of essential care (Stimpson et al. 2025). These meteorological variables are not independent but rather part of a unified physical-chemical continuum, in which one determines another. For example, during synoptic events such as temperature inversions or atmospheric blocking which affects relative humidity, suppressed boundary layer mixing can lead to substantial surface accumulation of particulate matter while ozone levels remain unaffected (Özdemir et al. 2024). This can travel through the airways and cause significant systemic inflammation through foreign bodies in the lungs, oxidative stress by generating free radicals, and cardiac and neurological issues by toxins entering the bloodstream (Anderson et al. 2012). The complexity of capturing atmospheric factors is highlighted elsewhere, with advanced analytical approaches required to integrate large-scale, multi-source datasets for assessment of physical-chemical continuums to simulate three-dimensional cloud radiance (Sun et al. 2026a).

Historically, traditional statistical approaches such as generalised additive models have been used to quantify associations between meteorological variables and health outcomes, advancing understanding of climate–health relationships (Gasparrini et al. 2015; Mora et al. 2017). However, the rapid expansion in the volume, resolution, and diversity of environmental datasets has introduced analytical challenges. Climate data are inherently high-dimensional, non-linear, and spatio-temporally complex, making them increasingly difficult to model effectively using conventional methods alone (Kazanskiy et al. 2025). Such challenges of modelling complex environmental and spatial data statistically are for example found in the context of flow field simulation, where static terrain refinement corrections highlight the necessity of addressing high-dimensional and spatially heterogeneous data (Xue et al. 2026). This has created a growing need for analytical approaches capable of integrating large-scale, multi-source datasets to support more accurate prediction and interpretation. As the impact of variables like air quality is conditional on other variables in a non-monotonic manner, issues like feature reduction and pre-specified interactions mean these synergistic relationships cannot be accurately mapped using solely statistics (De Marco et al. 2022; Latoń et al. 2025; Sun et al. 2026b). It then suggests that the very signals that drive weather-related health risks may be excluded from traditional models, and therefore more advances techniques are required.

Artificial intelligence (AI) methods, particularly machine learning and deep learning techniques, are increasingly being explored to address these challenges. These approaches have demonstrated improved forecasting accuracy, enhanced detection of extreme weather events, and greater capacity to model climate variability, especially when implemented as hybrid models combining physical and data-driven methods (Subramaniam et al. 2022). AI-assisted weather prediction systems have also been shown to improve downscaling and reduce systematic biases compared with traditional models (Waqas et al. 2025). Such capabilities make AI particularly well-suited to automatically handling the non-linear and spatio-temporal structures characteristic of meteorological data without the need for manual model specification, positioning it as a promising tool for environmental health research through more granular mapping of how specific cascading climate states trigger downstream morbidity and mortality risks through a domino effect (Adjei 2025; Li et al. 2026). Indeed, spatio-temporal structures can be effectively resolved by balancing multi-scale constraints in nudging-based downscaling, which allows for improved data resolution (He et al. 2026). Despite its potential, the application of AI to meteorological data within health research remains an emerging field with several unresolved challenges. AI models can be computationally intensive, requiring significant energy resources and contributing to greenhouse gas emissions and electronic waste (Chen et al. 2025; Ueda et al. 2024). Conversely, they may also reduce environmental impact through improved efficiency, optimisation, and more informed decision-making (Guo et al. 2025). It could further reduce this by detecting future disasters through factor identification, structural safety assessment of infrastructure, and disaster mechanism modelling, allowing for preventative measures to mitigate the severity of extreme weather events and the demand they place on healthcare (Hu et al. 2026; Li et al. 2026). Additionally, the inclusion and handling of demographic variables such as sex, age, and ethnicity are critical when developing predictive models, as failure to account for these factors can introduce bias and lead to inequitable performance, particularly in health-related applications (Nazer et al. 2023; Norori et al. 2021; Obermeyer et al. 2019). Some of these ‘black box’ issues can be addressed through explainable AI, retrospective methods for understanding complex data. Examples clarify relationships between meteorological drivers and air pollutants (Birinci et al. 2026), but the framework remains scarcely applied in this field (Cao et al. 2026; Frasca et al. 2024).

Although AI has demonstrated strong potential in meteorology and climate science, there is limited consolidated evidence on how these tools are being applied specifically to meteorological data within health research, and what benefits, limitations, and implications arise from their use in this context. This may relate to slower adoption and reporting of AI in human health applications, whereby deterministic causal relationships are drawn between climate patterns and health outcomes without validating underlying mechanisms, perhaps due to inconsistent frameworks. That stands in stark contrast with the rapid growth of AI in climate science, whereby weather prediction models using real-time data and factoring in uncertainty are increasingly based on machine learning, benchmarked against existing physics-based standards (Huang et al. 2026; Inam 2025). It thus represents a key gap, particularly as extreme weather events are increasing in frequency and intensity due to climate change (Kumar et al. 2023), which is now recognised as one of the greatest threats to global health and disproportionately affects socially and medically disadvantaged populations (The Lancet Respiratory Medicine 2025). Timely and reliable identification of weather-related health risks, and of populations most vulnerable to them, is therefore essential to prevent worsening health inequities. As such, this rapid review aims to synthesise recent studies involving AI for meteorological data applications in health research to better understand its use.

## Materials and methods

### Study design

A rapid review followed the Reporting Items for Systematic Reviews and Meta-Analyses extension for rapid reviews (PRISMA-RR) (App. 1). Rapid reviews are especially suitable for emerging research areas such as AI and environmental health where evidence is expanding rapidly, with immediate synthesis required to identify key trends and knowledge gaps before the artificial intelligence methods addressed become outdated. As opposed to full systematic reviews, quality appraisal of articles and registration of the protocol are not needed for rapid reviews, which ensures timely analysis (Stevens et al. 2025).

### Eligibility criteria

Peer-reviewed studies published in English between January 2020 and October 2025 were eligible for inclusion to capture recent applications of contemporary AI techniques, with searches performed on the 13^th^ of October 2025. Studies of any empirical design were included if they applied artificial intelligence tools to meteorological data in the context of human health research. Studies were excluded if they did not incorporate an AI component, meteorological data, participant characteristics including sample sizes, or human health outcomes. Non-peer-reviewed publications, including preprints, study protocols, commentaries, and conference abstracts, were also excluded. As AI is heterogeneously defined and applied, a prespecified terminology was not imposed on the papers, but rather articles were included when ‘artificial intelligence’ was explicitly stated regardless of what this was considered.

### Information sources and search strategy

Bibliographic databases searched included PubMed, Web of Science, and Scopus. Search terms were developed with the assistance of a climate change expert, combining free-text keywords and controlled vocabulary (MeSH). These focused on the following phenomena: 1) artificial intelligence, including the terms machine learning, deep learning, predictive modelling, language processing, Random Forest, gradient boosting, and autoencoder; 2) meteorological data, including the terms environmental, climate, weather, temperature, humidity, air quality, and geostat; 3) health outcomes, including the terms healthcare, disease, illness, morbidity, and hospital; and 4) empirical study, including observational study, experimental study, quasi-experimental study, secondary study, and quantitative study. The complete search strategy is available in the supplementary material (App. 2). Hand searches of Google Scholar were also included using the same terms, provided articles met eligibility criteria.

### Study selection

**Figure 1:**
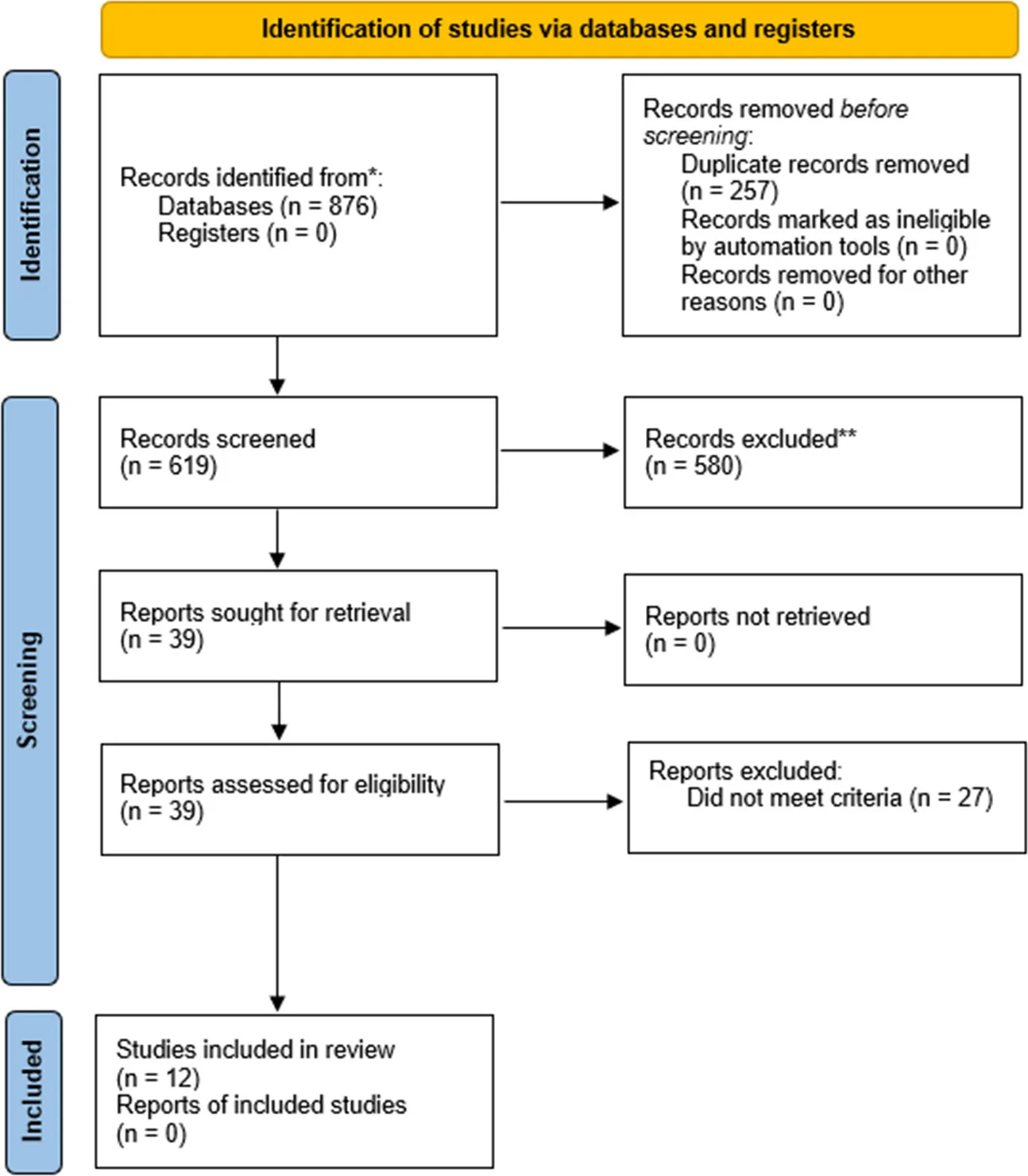
PRISMA flow diagram with numbers of records identified, screened, excluded, and included for studies which examine use of meteorological data in artificial intelligence tools to address health outcomes

Following duplicate removal, two reviewers (LVM and LS) independently screened all titles and abstracts against the eligibility criteria in Rayyan (Ouzzani et al. 2016). Full texts of potentially relevant articles were retrieved for detailed assessment, with disagreements resolved through discussion or consultation with a third reviewer (CG). The study selection process is illustrated in a PRISMA flow diagram outlining numbers of records identified, screened, excluded, and included (Fig. 1).

### Data extraction

A standardised data extraction form was developed. Extracted variables included author, publication year, country, objectives, study design, data sources, meteorological variables, health outcomes, AI tools applied, model performance metrics across stages, and reported findings and limitations. Data extraction was conducted independently by two reviewers (LVM and SG), following pilot extraction of three articles to ensure there was no missing or irrelevant information captured, with discrepancies resolved through discussion.

### Data synthesis

Due to heterogeneity in study aims, data sources, AI techniques, and outcome measures, narrative synthesis was conducted in line with the Synthesis Without Meta-analysis guidelines (Campbell et al. 2020). Findings were grouped by meteorological variable, health outcome, and AI technique. Methodological patterns, model performance, and gaps in evidence were summarised. All articles were described in each section of the narrative synthesis for consistency.

## Results

Using the eligibility criteria, 876 studies were identified of which 12 were included in this review (Fig. 1).

### Study characteristics

Most included studies were observational (*n* = 8), with fewer experimental designs (*n* = 2), alongside one longitudinal study [ID10] and one retrospective cohort study [ID11]. Study populations mainly comprised adults aged 18–65 years (*n* = 5) or children (*n *= 6); one study included both age groups [ID10], and one focused on adults aged ≥65 years [ID11]. Sex and age were reported in all studies, whereas ethnicity was reported in only one [ID11]. Sample sizes ranged from 12 to over 10 million participants (<1,000, *n* = 4; 1,000–10,000, *n* = 6; >100,000, *n* = 2). Data collection periods varied from 20 minutes to 11 years (mean: 5 years), with one study not reporting duration [ID4]. Six studies were conducted in high-income countries (United States, *n* = 2; France, Italy, Japan, and South Korea, *n* = 1 each), predominantly in temperate climates. The remaining studies were undertaken in middle- to low-income settings with extreme climates (Bangladesh, China, Kenya, Mali, and Vietnam; *n* = 1 each), which are generally more exposed to weather-related risks.

### Meteorological variables

The most common climate variable included was air quality including particulate matter (PM in 2.5 or 10 micrometres) and other pollutants, as well as temperature, including sunshine and thermal inversion (*n* = 6 each). Remaining studies addressed humidity and rainfall (*n* = 5), pressure and wind (*n* = 4), and seasonality (*n* = 2). To reconstruct these, ground stations or air sensors were used in all studies, with the majority also using satellite data (*n* = 7). How site-specific the measurements were depended on the study, ranging from local (*n* = 10) to regional [ID11] or national [ID8] (Table 1).

**Table 1:** Study characteristics for included studies which examine use of meteorological data in artificial intelligence tools to address health outcomes

**ID, author, year**	**Objective**	**Study design, population**	**Data sources, duration**	**Meteorological data included**	**Health conditions addressed**	**Artificial intelligence type used**
1. Alotaibi et al. 2023	Devise a personalised alert system tailored to individual lung function and environmental conditions to manage respiratory disease	Observational pilot study of young adults in Saudi Arabia (*n* = 12)	Pulmonary function tests, air quality sensor data collected over 20 minutes	Particulate matter, relative humidity, and ambient temperature	Chronic respiratory disease	Feature selection, linear regression, multilayer perceptron, support vector machine
2. Choi et al. 2024	Propose a classification model using personal biometric characteristics to identify individuals who are vulnerable to hot environments	Experimental study of men in South Korea (*n* = 70)	Environmental sources, wearable device data collected over 65 minutes	Air temperature, partial vapor pressure, mean radiant temperature, air velocity	Heat-related illness	Time-series clustering, k-means clustering
3. Czapar et al. 2024	Examine water source, rainfall, and sanitation in relation to the vaginal microbiome	Prospective observational cohort study of adolescent girls in Kenya (*n* = 436)	Satellite measurements, gauge data, vaginal swabs, self-report data collected over 18 months	Rainfall, water source	Bacterial vaginosis, vaginal community state	L0L2 and Lasso regression with bootstrapping
4. Garbern et al. 2022	Develop and externally validate the accuracy of a mobile software application to predict viral-only aetiology of acute diarrhoea	Prospective observational study of children in Bangladesh and Mali (*n* = 199)	Global Environmental Monitoring System, weather station data collected over unspecified time	Seasonality	Diarrhoea	Random Forest, pre- and post-test odds
5. Katsuki et al. 2023	Predict the number of hourly headaches from weather factors	Retrospective observational cross-sectional study of adults in Japan (*n* = 4 375)	Symptom application, Japan Meteorological Agency data collected over 12 months	Barometric pressure, humidity, temperature, rainfall	Migraine, headache	Time-series clustering, feed forward neural network, eXtreme Gradient Boosting
6. Maio et al. 2025	Investigate the association between long-term air pollution and COPD symptoms and diagnosis	Observational study of adults in Italy (*n* = 14 420)	Satellite observations, spatial(-temporal) data, self-report data collected over 6 years	PM10, PM2.5, NO2, mean summer concentrations of O3	COPD, dyspnoea, chronic cough/phlegm	Random Forest
7. Marquès et al. 2022	Assess the underlying effect of long-term exposure to NO2 and PM10 on the severity and mortality of COVID-19	Retrospective observational study of adults in Spain (*n* = 2 112)	Air monitoring cabins, hospital data collected over 6 years	NO2 and PM10	COVID-19	Random Forest, multivariate predictive models
8. Ming et al. 2022	Assess the validity of a prediction tool for whether children have dengue or febrile illness using seasonality	Prospective observational clinical study of children in Vietnam (*n* = 8 100)	Hospital records, world bank data collected over 30 days	Seasonality, temperature, average precipitation	Dengue, other febrile illness	Gradient boosting machine learning
9. Suarez Castillo et al. 2025	Examine the differential impacts of early childhood air pollution exposure on children’s healthcare	Quasi-experimental study of children in France (*n* ≈ 340 000)	Satellite measurements, emission inventories, hospital data collected over 10 years	Temporary increases in PM2.5, thermal inversions, wind direction	Bronchitis, obstructive airway disease	Generic machine learning
10. Su et al. 2024	Examine the association between daily air pollution and respiratory symptoms in participants with asthma and COPD using digital sensors monitoring inhaler use	Longitudinal study of children and adults in the United States (*n* = 3 386)	Digital sensors on inhalers for use and location, remote sensing of air quality, land use, land cover, vegetation, self-report data collected over 8 years	NO2, PM2.5, O3	Asthma, COPD	Deletion/substitution/addition modelling, Random Forest
11. Vega et al. 2025	Characterise the associations between PM2.5 exposure and cause-specific hospitalisations	Retrospective cohort study of 65+ year-olds in the United States (*n* = 10 369 361)	Ground measurements, satellite observations, reanalysis data sources, hospital data collected over 11 years	PM2.5 from wildfire smoke	Respiratory disease, cardiovascular disease, cancer, injury, blood disease, neuropsychiatric disorder, digestive system disease, genitourinary disease, endocrine disorder, infectious and parasitic disease, connective tissue and musculoskeletal diseases, nervous system disease, and skin and subcutaneous tissue disease	Distributed lag models with splines, time-series analysis
12. Wang et al. 2021	Assess the impact of meteorological indicators on pneumonia severity	Prospective observational study of children in China (*n* = 6 611)	Nasal swabs, surface stations, China Air Quality Online Monitoring and Analysis Platform data collected over 10 years	Air temperature, wind speed, atmospheric pressure, relative humidity, precipitation, sunshine hours, surface temperature, air quality, PM2.5, PM10, SO2, CO, NO2, O3	Pneumonia, viral codetections	Random Forest

### Health outcomes

Health outcomes amongst included studies focused predominantly on respiratory disease such as asthma and chronic obstructive pulmonary disease (COPD) (*n* = 6), as well as bacterial or viral illness like diarrhoea (*n* = 4). The remaining studies focused on heat-related illness [ID2], migraine and headaches [ID5], COVID-19 [ID7], febrile illness including dengue [ID8], and cardiovascular disease [ID11]. Only one study [ID11] focused on multiple long-term conditions. These were assessed through physiological tests (*n* = 5), hospital records (*n* = 4), or self-reports (*n* = 4) (Table 1, 2).

**Table 2:** Findings and limitations of included studies which examine use of meteorological data in artificial intelligence tools to address health outcomes

**ID, author, year**	**Model performance**	**Subgroup analysis**	**Key findings**	**Application to health outcomes**	**Reported study limitations**
1. Alotaibi et al. 2023	Correlation coefficient, root mean squared error	Yes (environmental conditions)	Different environments elicit different pulmonary responses, allowing for predictive alerts for mitigation of asthma symptoms	Pulmonary response to different air quality	Low sample size, no extreme environmental variations, short study duration, healthy individuals
2. Choi et al. 2024	Overfitting, sensitivity, specificity, recall, precision, area under the receiver operating curve	Yes (vulnerability)	Higher metabolic rate, related to BMI, body composition, heart rate, and body temperature, associated with higher vulnerability to heat strain	Heat strain vulnerability based on physiological factors	Experimental conditions not necessarily reflective of real world
3. Czapar et al. 2024	Not specified	Yes (water source, latrine type, study visit)	Increased rainfall and streaming water associated with decreased risk of vaginal bacteria	Impact of rainfall and water streaming on vaginal bacteria	No water samples
4. Garbern et al. 2022	Area under the curve, specificity, sensitivity	Yes (country)	Incorporation of seasonality aids in accurate identification of high likelihood of viral-only diarrhoea using machine learning	Impact of seasonality on viral-only diarrhoea	Limited generalisability, short study duration, no standardised thresholds
5. Katsuki et al. 2023	Sensitivity, validation, feature importance	Yes (environmental conditions, age, sex, season, location)	Low barometric pressure, barometric pressure changes, higher humidity, and rainfall were associated with an increased number of headache occurrences	Impact of air pressure, humidity and rainfall on headaches	Selection bias, no determination of best predictive model, no calibration
6. Maio et al. 2025	Not specified	Yes (smoking)	Increases in PM10, PM2.5, and NO2 increased COPD and dyspnoea diagnosis and symptoms	Impact of air particles on COPD and dyspnoea diagnosis and symptoms	No lung function testing, occupational exposure not considered, no recent data
7. Marquès et al. 2022	Not specified	No	PM exposure increased COVID-19 severity and mortality	Impact of air particles and COVID-19	No PM exposure per severity group, assumes people live near admission hospital
8. Ming et al. 2022	Area under the receiver operating curve, specificity, sensitivity, predictive value, Brier score	No	Seasonality affects febrile conditions and thereby model performance, while inclusion of climate variables did not affect model performance	Seasonality and febrile conditions	Selection bias, no localised weather, misclassification, lack patient data
9. Suarez Castillo et al. 2025	Average effects, heterogeneity analysis, multicollinearity, robustness	Yes (thermal inversion, wind direction, vulnerability, income, premature birth, exposure)	Hospital emergency admission and drug delivery use for respiratory disease are higher during surges of air pollution, particularly in children with poor health indicators at birth	Health service usage for respiratory disease during air pollution	Not specified
10. Su et al. 2024	Predictive capability, sensitivity	No	Increased air pollution links with increased inhaler use for respiratory symptoms, especially with multiple pollutants	Air pollution and inhaler use	Selection bias, limited generalisability, limited location information, lack potential confounders
11. Vega et al. 2025	Not specified	No (despite different ethnicities)	Daily county-level unscheduled hospitalisations for respiratory concerns increased from same-day and preceding week smoke, and for cardiovascular disease non-statistically	Wild-fires and hospital admission	Exposure measurement error, residual confounding, no recent data or high concentrations
12. Wang et al. 2021	Area under the curve, prediction efficiency	No	Weather and pollution increased pneumonia severity, which associated with viral codetections	Weather and pollution impact on pneumonia severity	Data bias, limited generalisability

### Artificial intelligence tools

The most commonly used form of AI was decision trees including Random Forest (*n* = 5), followed by time-series or clustering analysis (*n* = 3), regression (*n* = 2), gradient boosting (*n* = 2), and multivariate/-layer analysis (*n* = 2). The remainder utilised support vector machine [ID1], feature selection [ID1], neural networks [ID5], deletion/substitution/addition analysis [ID10], or unspecified machine learning [ID9]. Performance metrics were not specified in four studies. When included, most assessed model sensitivity (*n* = 5). Area under the (receiving operator) curve (*n* = 4), specificity (*n* = 3), and predictive capability or efficiency (*n* = 3) were also assessed. The remainder utilised correlation coefficient [ID1], root mean squared error [ID1], precision [ID2], overfitting [ID2], recall [ID2], validation [ID5], feature importance [ID5], Brier score [ID8], multicollinearity [ID9], robustness [ID9], and heterogeneity [ID9] (Table 1, 2).

Reasoning behind the choice of each AI tool and performance metric included was often lacking (*n* = 7), with an underrepresentation of explainable AI (only in [ID5]), and therefore the added benefit of using machine learning compared to statistical techniques was generally unclear but likely related to the size and complexity of datasets. Indeed, when reported, justifications included the use of clustering to help identify vulnerable individuals [ID2] or find and understand patterns in health data (in [ID5], with no justification for remaining models used here), of regression to help select risk predictors in low signal-to-noise settings [ID3], of decision trees to help assess relative importance of pollution against other factors [ID7] or identify determinants and patients per virus [ID12], of mixed models for flexibility and consistency with previous work on disease aetiology [ID4], and of generic machine learning for unspecified computational reasons and to address heterogeneity or imbalances between groups [ID9]. The aforementioned ability of AI to process non-linear relationships and yield accurate predictions was highlighted throughout (especially in [ID12]) (Table 2).

### Reported findings

AI mostly played a role in the determination of aetiological factors of health outcomes studied (*n* = 8), followed by their diagnosis [ID8] or management [ID1], as well as identification of affected individuals [ID2] and prediction of prevalence [ID12]. Subgroup analysis was performed in 7 out of 12 studies, predominantly comparing patient data across environmental conditions (*n* = 4) or comparing those with the disease in question or risk factors thereof with less vulnerable individuals (*n* = 3) to reveal additional insights that aid the model’s predictive value. This was often categorised through AI, using feature selection [ID1], clustering [ID2,5], regression [ID3], and heterogeneity analysis [ID9], but also manually based on known associations [ID3,6] or available data [ID4,5].

More specifically, studies using Random Forest identified that incorporation of air pollution and seasonality improved the predictive value of their model, as exposure to extremes linked to increased prevalence and severity of diarrhoea [ID4], dyspnoea [ID6], COVID-19 [ID7], asthma [ID10], and pneumonia [ID12]. Studies using time-series analysis and gradient boosting further identified that incorporation of temperature, humidity, air pressure, wildfire smoke, and seasonality also improved predictive value of their model, as exposure to extremes linked to increased prevalence and severity in respiratory and cardiovascular or heat-related disease [ID2,11], headaches [ID5], and febrile conditions [ID8]. Lastly, studies using regression or unspecified machine learning also identified that incorporation of rainfall, pollution, and temperature improved the predictive value of their model, as exposure to extremes linked to increased prevalence and severity of bronchitis [ID1,9] and vaginal bacteria [ID3]. As such, there was no clear association between AI technique used, meteorological variable integrated, and health outcome addressed, except for between pollution and respiratory disease (Table 2).

### Suggested limitations

Limitations were reported in 11 out of 12 studies (not in [ID9]). These predominantly involved data issues (*n* = 8), including selection bias in three studies, absence of recent or localised data in three studies, and lack of standards [ID4], misclassification [ID8], and exposure measurement error [ID11] in one study each. Furthermore, methodological steps were missed (*n* = 4), including lack of calibration [ID5], model determination [ID5], tests [ID6], subgroup analysis [ID7], and consideration of confounders [ID10]. Limited generalisability has also been recorded (*n* = 3), and experiments are described as not reflecting the real world due to the absence of extreme variations or multiple influencing factors (*n* = 3). Lastly, a short study duration (in [ID1,4] reflecting less than half an hour) and low sample size (in [ID1], reflecting a dozen healthy participants to predict asthma) has been identified (Table 2).

## Discussion

This rapid review aimed to synthesise evidence from recent studies using AI for meteorological data applications in health research to better understand its use. Twelve articles published between 2020 and 2025 met eligibility criteria. Most were focused on utilising air quality and temperature data to predict the prevalence and severity of respiratory or bacterial diseases, with other work focusing on disease management, vulnerability estimations, and healthcare utilisation. AI tools predominantly involved Random Forest and time-series or clustering analysis, mostly assessed through sensitivity, yet the reasoning behind the choice of AI tool or performance metric was largely unreported and the added benefit of using AI compared to traditional statistical techniques was not made explicit as explainable AI techniques were lacking. Notably, time-series analysis, regression, and pre- or post-odds can function as statistical rather than machine learning approaches, yet in all included studies these were specified as involving artificial intelligence despite missing details. Furthermore, such classification metrics, regression metrics, and time-series metrics are appropriate at different stages of the model application. While that was expected to determine the choice, this is not clearly described in included papers, hence its grouping here despite incomparability across tasks.

The low number of studies extracted reflects of a lack of participant characteristics in studies utilising AI and meteorological data in health settings, meaning they did not meet inclusion criteria, rather than a lack of studies themselves. Most of the excluded research utilised medical records without recording the sample size, and most of the included research showed an absence of ethnicity data in model building which likely leads to decreased performance. Existing health inequity may also be exacerbated by underrepresentation of those most vulnerable to weather-induced health risks, including adults aged ≥65 years and individuals living with mental health or long-term conditions, although children were considered in half the studies. Additionally, high-income countries with temperate climate are less likely to be impacted by climate change than low-income countries with extreme climates, and as such their overrepresentation may affect generalisability. While findings indicate that incorporation of meteorological data can improve prediction of health outcomes, these methodological and data issues limit the comparability and reproducibility of such AI studies.

### Comparison to existing literature

Our findings are in line with previous work. This indeed reveals that climate change has a larger impact on countries with extreme compared to temperate climates, as well as on low- compared to high-income countries, as these both contribute and adapt to greenhouse gases to a lesser extent while experiencing accelerated population growth (Gilli et al. 2024; Herold et al. 2017; Hosseinzadehtalaei et al. 2025). One review also discussed the benefits and drawbacks of AI in environmental health, highlighting data heterogeneity, scalability, model accuracy, algorithmic bias, and ethical issues as obstacles. While it placed this in the context of low-income countries and health inequity, it did not provide any participant characteristics for tool development including sample sizes (Adjei 2025). Another systematic review focused on utilising machine learning to predict health risks associated with extreme weather conditions, particularly mortality, heat-related illness, and post-traumatic stress disorder. This too highlighted a low number of studies as well as the need for better quality data, development of data standards, and consideration of bias and health inequity. However, meteorological data other than extreme weather, such as the link between pollution and respiratory disease which is one of the main themes here, had not been addressed and recent articles were missing (Ssebyala et al. 2024).

The inclusion of air pollution in this review draws out the potential for targeted AI-driven interventions to reduce the number of premature deaths it is known to induce. Indeed, a recent governmental report found that climate adaptation efforts for health are insufficient in the UK (Government UK 2025), requiring both short-term and long-term adaptation and mitigation strategies (Ofremu et al. 2025). This review also draws out the potential for AI tools to predict service demand and medication usage during extreme weather conditions, which could lead to improved planning of healthcare capacity and resource management tailored to specific climate events (Bari et al. 2023). However, sustainable practices must be employed to ensure that AI for meteorological data applications in health research mitigates rather than exacerbates climate change, and as such weather-induced health risks. A systematic review of the role of AI in achieving the sustainable development goals such as those from the United Nations highlighted that research social-oriented goals receive less attention than infrastructure-oriented goals, especially in low-income countries, and calls for more inclusive international collaboration with more ethical reflection of AI (Gao et al. 2026). An interrogation of how the inclusion of AI’s environmental effects reshape ethics frameworks in global health confirmed the need for improved transparency in model building, as suggested in this work too (Collins et al. 2015; Fiske et al. 2025). Explainable AI techniques such as Shapley additive explanations (SHAP), local interpretable model-agnostic explanations (LIME), partial dependence plots, permutation feature importance, and explainable boosting machines may help with this by improving transparency (Birinci et al. 2026; Cao et al. 2026; Frasca et al. 2024; Inam 2025).

### Strengths and limitations

This review provides the first comprehensive synthesis of recent studies combining various types of meteorological data, artificial intelligence, and health research. Strengths include the use of a transparent and systematic approach guided by PRISMA-RR methodology, the inclusion of dual independent screening and data extraction, and structured narrative synthesis across major databases. By focusing on studies published between January 2020 and October 2025, the most up-to-date developments in AI are captured, thereby ensuring that findings reflect contemporary computational capabilities. It further highlights the diversity of AI techniques, meteorological variables, and health applications, providing valuable insight into how machine learning is being operationalised for different diseases. However, the rapid review design, while enabling timely synthesis of an emerging field, limits the breadth of database coverage and depth of formal quality appraisal. Restricting our inclusion criteria to English and peer-reviewed publications may have introduced language and publication bias, while the heterogeneity of methods and data precludes quantitative meta-analysis.

### Conclusions

The escalating threat of climate change to morbidity and mortality underscores the urgent need for timely, accurate, and population-specific health predictions. This rapid review demonstrates that artificial intelligence has significant potential to support adaptive and mitigative action, by enhancing predictive models that identify individuals most at risk of negative weather-induced health outcomes. Such improvements could enable targeted interventions to prevent hospitalisation and premature death. However, the exclusion of vulnerable groups and limited reporting of participant characteristics, combined with a lack of clear model justification, undermine the robustness and reliability of such tools. Addressing these gaps will require stronger guidelines and greater methodological rigour in future research that applies AI modelling of meteorological variables to improve health outcomes, with greater application of explainable AI.

There are several concrete steps recommended to achieve this. First, integrating frameworks like SHAP or LIME will help unpack ‘black box’ models and transparently map non-linear interactions between meteorological variables and health. Enforcing the reporting of age, sex, ethnicity, and socioeconomic status will prevent algorithmic bias and protect vulnerable populations, supported by enforcing the reporting of clear rationales for the selection of specific machine learning tools and performance metrics to ensure appropriateness for the dataset. Lastly, combining energy-efficient AI designs with real-time climate data and physics-based standards will enhance causal validity and ensure that environmental health modelling does not worsen weather-induced risks.

## Data Availability

All articles utilised are publicly available.

## Appendices

### Appendix 1

PRISMA Checklist for the presented review of studies which examine use of meteorological data in artificial intelligence tools to address health outcomes

**Table:** Preferred Reporting Items for Systematic reviews and Meta-Analyses extension for Scoping Reviews (PRISMA-ScR) Checklist

SECTION	ITEM	PRISMA-ScR CHECKLIST ITEM	REPORTED ON PAGE #
TITLE			
Title	1	Identify the report as a scoping review.	1
ABSTRACT			
Structured summary	2	Provide a structured summary that includes (as applicable): background, objectives, eligibility criteria, sources of evidence, charting methods, results, and conclusions that relate to the review questions and objectives.	2
INTRODUCTION			
Rationale	3	Describe the rationale for the review in the context of what is already known. Explain why the review questions/objectives lend themselves to a scoping review approach.	3-4
Objectives	4	Provide an explicit statement of the questions and objectives being addressed with reference to their key elements (e.g., population or participants, concepts, and context) or other relevant key elements used to conceptualize the review questions and/or objectives.	4
METHODS			
Protocol and registration	5	Indicate whether a review protocol exists; state if and where it can be accessed (e.g., a Web address); and if available, provide registration information, including the registration number.	4
Eligibility criteria	6	Specify characteristics of the sources of evidence used as eligibility criteria (e.g., years considered, language, and publication status), and provide a rationale.	4-5
Information sources*	7	Describe all information sources in the search (e.g., databases with dates of coverage and contact with authors to identify additional sources), as well as the date the most recent search was executed.	5
Search	8	Present the full electronic search strategy for at least 1 database, including any limits used, such that it could be repeated.	5, 23
Selection of sources of evidence†	9	State the process for selecting sources of evidence (i.e., screening and eligibility) included in the scoping review.	5
Data charting process‡	10	Describe the methods of charting data from the included sources of evidence (e.g., calibrated forms or forms that have been tested by the team before their use, and whether data charting was done independently or in duplicate) and any processes for obtaining and confirming data from investigators.	5
Data items	11	List and define all variables for which data were sought and any assumptions and simplifications made.	5
Critical appraisal of individual sources of evidence§	12	If done, provide a rationale for conducting a critical appraisal of included sources of evidence; describe the methods used and how this information was used in any data synthesis (if appropriate).	n/a
Synthesis of results	13	Describe the methods of handling and summarizing the data that were charted.	6
RESULTS			
Selection of sources of evidence	14	Give numbers of sources of evidence screened, assessed for eligibility, and included in the review, with reasons for exclusions at each stage, ideally using a flow diagram.	6-7
Characteristics of sources of evidence	15	For each source of evidence, present characteristics for which data were charted and provide the citations.	7-8, 11
Critical appraisal within sources of evidence	16	If done, present data on critical appraisal of included sources of evidence (see item 12).	n/a
Results of individual sources of evidence	17	For each included source of evidence, present the relevant data that were charted that relate to the review questions and objectives.	8-13
Synthesis of results	18	Summarize and/or present the charting results as they relate to the review questions and objectives.	8-13
DISCUSSION			
Summary of evidence	19	Summarize the main results (including an overview of concepts, themes, and types of evidence available), link to the review questions and objectives, and consider the relevance to key groups.	14
Limitations	20	Discuss the limitations of the scoping review process.	15
Conclusions	21	Provide a general interpretation of the results with respect to the review questions and objectives, as well as potential implications and/or next steps.	16
FUNDING			
Funding	22	Describe sources of funding for the included sources of evidence, as well as sources of funding for the scoping review. Describe the role of the funders of the scoping review.	16

JBI = Joanna Briggs Institute; PRISMA-ScR = Preferred Reporting Items for Systematic reviews and Meta-Analyses extension for Scoping Reviews.

* Where *sources of evidence* (see second footnote) are compiled from, such as bibliographic databases, social media platforms, and Web sites.

† A more inclusive/heterogeneous term used to account for the different types of evidence or data sources (e.g., quantitative and/or qualitative research, expert opinion, and policy documents) that may be eligible in a scoping review as opposed to only studies. This is not to be confused with *information sources* (see first footnote).

‡ The frameworks by Arksey and O’Malley (6) and Levac and colleagues (7) and the JBI guidance (4, 5) refer to the process of data extraction in a scoping review as data charting*.*

§ The process of systematically examining research evidence to assess its validity, results, and relevance before using it to inform a decision. This term is used for items 12 and 19 instead of "risk of bias" (which is more applicable to systematic reviews of interventions) to include and acknowledge the various sources of evidence that may be used in a scoping review (e.g., quantitative and/or qualitative research, expert opinion, and policy document).

*In absence of a developed rapid review checklist, the scoping review checklist was utilised, from:* Tricco AC, Lillie E, Zarin W, O'Brien KK, Colquhoun H, Levac D, et al. PRISMA Extension for Scoping Reviews (PRISMA-ScR): Checklist and Explanation. *Ann Intern Med*. 2018;169:467–473. https://doi.org/10.7326/M18-0850.

### Appendix 2

**Table:** Search terms to systematically identify studies which examine use of meteorological data in artificial intelligence tools to address health outcomes

MeSH and Free Text Search Terms	Filters/Refined by	Databases
((“artificial intelligence” OR “machine learning” OR “deep learning” OR “prediction model” OR “predictive model” OR “predictive modelling” OR “language processing” OR “autoencoder” OR “Random forest” OR “gradient boosting”) AND (“meteorology” OR “meteorological” OR “environmental” OR “climate” OR “weather” OR “temperature” OR “humidity” OR “air quality” OR “forecast” OR “forecasting” OR “nowcast” OR “nowcasting” OR “geospatial” OR “geo-spatial” OR “geostat” OR “geo-stat”) AND (“health” OR “healthcare” OR “disease” OR “illness” OR “morbidity” OR “hospital” OR “epidemiology”) AND (“empirical study” OR “observational study” OR “experimental study” OR “quasi-experimental study” OR “secondary study” OR “quantitative study”))	Restricted to the English language	PubMed (*n* = 55 before screening)
		Web of Science (*n* = 407 before screening)
		Scopus (*n* = 414 before screening)
